# The “Great Lockdown” and cultural consumption in the UK

**DOI:** 10.1007/s10824-022-09463-6

**Published:** 2022-11-17

**Authors:** Hasan Bakhshi, Salvatore Di Novo, Giorgio Fazio

**Affiliations:** 1 Nesta, Creative Industries Policy and Evidence Centre (PEC), 58 Victoria Embankment, London, EC4Y 0DS UK; 2 Newcastle University, 5 Barrack Road, Newcastle upon Tyne, NE1 4SE UK; 3 SEAS, University of Palermo, Palermo, Italy

**Keywords:** COVID-19, Lockdown, Cultural participation, Survey data, D1, Z1, C25

## Abstract

In this paper, we exploit a unique weekly longitudinal survey of adults in the UK purposefully collected to study consumption choices with respect to cultural content types during the first Covid-19 national lockdown (the “Great Lockdown”). We look for changes in the probability of consuming different cultural and creative types of content (Music, Movies, TV, Games, Books, Magazines and Audiobooks), as well as changes in the overall variety of consumption. We find that changes in consumption depend on the type of content. In particular, other things being equal, the likelihood of listening to Music and playing Games went up and the likelihood of reading Books went down. We find little statistically significant evidence of changes in the probability of consumption of the other types of content. We find that, while on average individuals increased the variety of their consumption, the statistical significance of this increase varied depending on the socio-demographic and economic characteristic of interest. In particular, we find evidence of an increase in the variety of consumption for those at the bottom of the distribution of socio-economic status, which speaks to the importance of access to culture and creativity during lockdown for this specific social class.

## Introduction

The Covid-19 pandemic is the largest peacetime health and economic shock in a generation. As governments around the world implemented Non-Pharmaceutical Interventions (NPIs), such as social distancing and Shelter in Place (SiP) orders, to slow the spreading of the disease, entire parts of the economy had to be shut down. Public intervention had to step up to keep the economy afloat.^1^ In the UK, the pandemic has hit hard parts of the Cultural and Creative Industries (CCIs) at a time when household expenditure had been growing fast, as shown by data from the Office for National Statistics (ONS).^2^

On the production side, while some parts of the sector have been heavily damaged, the CCIs have also shown great flexibility and resilience, albeit with unprecedentedly large support packages from the Government. The forced lack of “in-person” consumption has also accelerated the trends in digitisation across all forms of cultural and creative content types (CCCs) that were already present pre-pandemic.^3^ Several organisations quickly responded by moving their content online,^4^ initially, as a way to support people forced to stay at home and, later, as a new way to engage consumers.^5^ Such ability to “digitally” adjust, however, varies across sub-sectors and firms, consumers and content. Arguably, firms in sectors like games and publishing may have even seen their activities boosted by SiP measures compared with those reliant on physical presence, which, instead, have been forced to dramatically rethink their operating and business models to avoid shutting down. Similarly, the ability to digitally adjust may have differentially affected cultural organizations in relation to their geographical location and audience spectrum, as discussed by Holcombe-James (2021) for Australia.

On the consumption side, the pandemic-induced recession has caused a large fall in income and employment in the overall economy, which, as noted above, would have been even larger without the intervention of governments around the globe. However, the shock, and the mitigating measures, may have not been felt identically by all consumers. For example, those working from home have been able to make economies in commuting time; workers in job retention schemes have seen a reduction in income but also a large increase in time available for non-work-related purposes, and working families with children have had to juggle between home-working and parental care. At the same time, heterogeneity in the technologies of consumption may have meant that these shocks to income and time may have played out differently in different cultural and creative activities. Hence, while the pandemic has represented a common massive shock, it may well have affected different consumers and types of CCC differently, conceivably in line with pre-existing inequalities across socio-economic status, occupation, gender and age. In this respect, this paper also links with the agent-based theoretical framework developed in Biondo et al. (2022), who explore the role of socio-economic standing (income and education) on the consumption (and production) of high vs lowbrow cultural activities.

Assessing whether shifts in consumption occurred and, if so, across which cohorts of consumers, is important for several reasons. First, from a simple welfare standpoint, it is critical to gauge whether and how the pandemic has affected people.^6^ Second, as the epidemic moves from pandemic to endemic, some foresight may come from these shifts on what the “new normal” could look like, which is critical for producers and distributors of CCCs who need to adapt their business models.^7^ Third, since the resilience of the CCIs and consumers also reflects unprecedented cash injections from the Government, such as the UK’s Cultural Recovery Fund for producers and income support policies for workers and self-employed, understanding such changes is essential for policymakers and policy organisations who need to devise cultural policies for the post-pandemic recovery, as the effects of these policy instruments wear off.

In the UK, the pandemic has triggered several studies on its effects on the cultural and creative industries, including many data collection exercises.^8^ In particular, studies by the UK Creative Industries Policy and Evidence Centre^9^ and The Audience Agency^10^ highlight the importance of cultural consumption during the pandemic for well-being and document an increase in consumption linked to the shift from physical to digital consumption. Some of these reports show that, following the removal of social distancing measures, some of the digital habits acquired during lockdown have persisted, together with an increase in engagement with local cultural consumption. While these studies provide useful descriptive evidence, multivariate analysis is needed in order to assess whether the pandemic affected cultural consumption and to what extent its effects differed across consumers.

Together with Feder et al. (2021), to the best of our knowledge, this paper represents one of the first comprehensive microeconometric studies of how cultural consumption has changed during the UK lockdown and its determinants.^11^ Moreover, against the backdrop of an increase in the availability of longitudinal data sets on traditional and in-person forms of cultural consumption (Ateca-Amestoy et al., 2017), critically, this paper also adds to the evidence on “remote” and online forms of consumption. In this respect, this paper also links with Aguiar et al. (2021), who study the effect of shifts in time availability on leisure demand for US individuals, following the Great Recession, finding a disproportionate increase in online recreation activities, like Games, for younger individuals—most notably men.

Hence, with the above in mind, in this paper we focus on the effects of the pandemic on the consumption side of the CCIs. The objective is to study whether and how consumers have changed their consumption with CCCs during the Covid-19 pandemic, specifically during the first national lockdown in the Spring of 2020. We refer to this as the “Great Lockdown” as this was the period when the strictest social distancing measures were in place throughout the UK.^12^ These required people to stay at home, except in the case of essential workers, businesses and venues to close in general, and for parents and carers to homeschool their children except again in the case of children or wards of essential workers.^13^

To this end, we exploit a unique nationally representative survey, conducted by the Creative Industries Policy and Evidence Centre in collaboration with the UK Government’s Intellectual Property Office and the research agency AudienceNet, to investigate consumers’ decisions with respect to home and online consumption of Music, Movies, TV, Games, Books, Magazines and Audiobooks and see how these changed over six consecutive weeks of lockdown.

Specifically, we look at how consumption changed during lockdown compared with the period prior to introduction of SiP measures for each of the seven above-mentioned types of content and for the variety of content types consumed. While the first part of the analysis tells us how patterns of consumption shifted for each different category of CCCs, the second allows inference on how cultural consumption as a whole changed and also whether consumers responded to being home-bound by seeking access to a greater variety of CCCs or, on the contrary, restricted their cultural consumption basket. Indeed, greater variety of choice tends to be associated with higher well-being in consumer research (see, for example, Mogilner et al., 2008 and Etkin & Mogilner, 2016). Etkin and Mogilner (2016), for example, find that filling a period of time like a day with a greater variety of activities increases happiness (albeit filling a much shorter period with greater variety may actually decrease it). Also in economics, greater variety of choice is associated with greater utility (see, for example, economics approaches where love-of-variety enters the utility function, as in Dixit and Stiglitz 1975).^14^ In this context, it is interesting to see whether and how the variety of cultural consumption shifted in lockdown, and if any of such effects varied across types of individuals, depending on their age, gender, living arrangements, employment and socio-economic status.

Having controlled for these observed socio-demographic and economic characteristics (SDEs), we show that the likelihood of changes in consumption depended on the type of CCC in question. In particular, we find that the probability of consuming Music and Games increased, and the probability of consuming Books decreased. We also find that the variety of CCCs engaged with went up. The association between consumption decisions and the socio-demographic characteristics of respondents, irrespective of SiP measures, is also content specific.

We also find that shifts in consumption during lockdown compared with the pre-lockdown period varied significantly across the above-mentioned individual characteristics. In terms of employment status, for example, statistically significant changes were observed for the Employed and the Retired, who increased their access to Music and Games and reduced their consumption of Books. Perhaps surprisingly—given they are the oldest—the Retired displayed the largest increase in Games consumption. Among the other occupational categories, the only other significant shifts pertained to the Unemployed for whom other things being equal, the likelihood of playing Games doubled in lockdown.

In contrast to Games, we find that the increase in Music consumption was more pronounced among the younger cohort (and especially the Employed). The overall decrease in the probability of Books consumption is accounted for by those individuals living with others, e.g. those living with a partner or parents, older cohorts and by those above the bottom quartile of the socio-economic status distribution (see Sects. 5.1 and 5.2 for the full analysis). We find some partial evidence that the pre-existing differences in consumption due to socio-economic status were altered in the lockdown. In particular, higher socio-economic status was associated with lower Books consumption and lower socio-economic status was associated with higher Games consumption. In addition, in general, consumption variety increased for those in the lowest socio-economic status quartile. This is in contrast to the suggestion by other studies (e.g. Feder et al., 2021) that digital engagement in lockdown if anything reinforced existing differences in cultural consumption by different socioeconomic groups.

The rest of the paper is organized as follows. The next section gives a brief and non-exhaustive account of some of the key papers in the relevant economics literature on cultural consumption. Section 3 provides information on the data and the definition of variables used in our analysis. Section 4 assesses changes in cultural consumption following the national restrictions introduced in the UK towards the end of March 2020, and Sect. 5 discusses how these shifts in consumption vary by SDEs for each content category and the overall variety of CCCs consumption. Section 6 summarises the results and concludes.

## Related literature: determinants of cultural consumption and the impact of COVID-19

The literature on the consumption of cultural and creative content is vast and summarising it here would be beyond the scope of the paper.^15^ Instead, we provide a brief account of the literature that is more related to our study, with the objective of giving some context for the analysis and highlighting our contribution. In particular, we look at the determinants of cultural consumption in the pre-pandemic literature, focusing first on the literature looking at the role of socio-economic status and, then, at the literature on demographic differences in cultural consumption. Finally, we summarise the literature on the impact of Covid-19 on income and time use, and in particular those contributions that can help understand its heterogeneity across individuals based on their characteristics, which is the objective of the paper.

### Cultural consumption and socio-economic status

Economic studies on cultural consumption and its relationship with socio-economic status date back at least to Baumol and Bowen (1966) who document the *elitist* nature of cultural attendance. More recent contributions, like Borgonovi (2004), Seaman (2005) and Falk and Katz-Gerro (2016) mostly confirm this finding: better educated and richer individuals are more likely to include cultural activities in their consumption basket.^16^

The relationship between cultural consumption and inequality is also at the centre of a large literature focusing on social class. Heterogeneity in tastes across the social ladder—featuring, yet not limited to, so-called highbrow and lowbrow activities, has been, in particular, discussed in an extensive sociological literature starting from the seminal work of Bourdieu (1984) and onward, as individuals tend to keep their consumption habits in line with their close environment.

However, some studies have argued that individuals from higher social backgrounds have started to increasingly consume a mix of both highbrow and lowbrow culture, embracing “emerging” and “cosmopolitan” culture, something referred to as *cultural omnivorousness*. Using a survey of British comedy tastes, Friedman (2012) finds that omnivorousness is a feature of upwardly mobile individuals. In line with Bourdieu, individuals have lowbrow tastes when young, but acquire highbrow tastes if and as they age and progress towards higher social status. In turn, these findings align with the recent—lockdown-specific—theoretical predictions offered by Biondo et al. (2022). Furthermore, their findings of lowbrow activities being more easily added to the cultural consumption basket than high-brown counterparts, could have additional implication for highbrow activities, with the latter more likely to be adversely affected by shocks like the recent pandemic—and, more generally, in a context featuring limitations to individuals’ mobility and in-person interactions.

The previously mentioned trends in digitisation have clearly raised the question of whether digital technologies reduce or increase inequality and elitism in cultural consumption. On the one hand, digital media can represent a democratising force insofar as they can, in general, lower the cost of access. On the other, they can reinforce existing tendencies giving rise to digital omnivores, or indeed exacerbate pre-existing inequalities, if, for example, digital culture is less accessible for those without a reliable broadband connection, essential equipment or the required technological knowledge: all of which we would expect to correlate with lower income and/or less advantaged socio-economic groups.

In this direction, Mihelj et al. (2019) look at the role of digital media in driving cultural consumption and the ‘cultural divide’. They investigate this issue using the Taking Part Survey in England with a focus on museums and galleries and find that, on the one hand, digital media enhance consumption, both online and offline, but, on the other, inequality in consumption persists and, indeed, the gaps seem wider for online consumption than analogue consumption. Similarly, Weingartner (2020) also finds that inequalities in the analogue world are reproduced or even amplified in the digital world.

### Demographic factors: gender, household interactions and age

Gender, household interactions and age have also been acknowledged as determinants of cultural consumption. In general, women are found to be more likely to engage—both more intensively and more frequently—than men. Gender differences in cultural attendance may be related to differences in time allocation within the household (see, for instance, Browning et al., 1994) and/or to differences in workforce participation (Cellini & Cuccia, 2021).

Recent literature has also looked at household interactions and complementarities within the couple. Along these lines, Lazzaro and Frateschi (2017) use Italian survey data and investigate how the characteristics of individuals (like educational attainment) and couples (like the presence of children in the household) are related to arts participation. The authors document that the characteristics of the partner influence the couple’s participation in a broad set of cultural activities. Additionally, Mauri and Wolf (2020) provide evidence that increased bargaining power of women within households positively affects couples’ involvement in “women-dominated” cultural activities (such as ballet and opera).

The literature has also found a positive association between cultural participation and age. This is mainly rationalised in terms of “learning-by-consuming” and “habit-formation” phenomena, which have their foundations in the early contributions of Stigler and Becker (1977) and Becker and Murphy (1988). However, this evidence is not conclusive and some studies also find that younger cohorts are other things being equal more likely to attend theatre and ballet (see, for example, Borgonovi, 2004).

In this paper, we report overall evidence in line with the latter, but we expand it to consider also the effects of the Covid-19 pandemic. We find that other things equal, the younger cohorts are, in normal times, more likely to consume the set of CCCs considered. Following the implementation of SiP measures, however, we find interesting evidence that older cohorts became relatively more likely to consume certain types of content, such as Games.

It should be pointed out that the literature takes different perspectives on what actually should be considered as cultural consumption, which could have implications for the role of age and gender. Following Favaro and Frateschi (2007), the two most commonly used metrics pertain to the physical attendance of live performances and consumption of media (see, again, Borgonovi, 2004). In this paper, we mostly refer to the latter, although remote consumption is not restricted to “non-live” activities but, differently from the past, also extends to live-streamed events. Indeed, the focus of the existing cultural economics literature has mainly been on activities like ballet, cinema, music, opera and theatre, reflecting—to some extent—a bias towards live in-person events.^17^ One of the contributions of this paper, therefore, is also to provide evidence on a broader set of CCCs compared with past studies.

With respect to a sub-set of CCCs investigated in this study, Borowiecki and Prieto-Rodriguez (2015) and Borowiecki and Bakhshi (2018) provide evidence on (offline and online) videogames consumption for Spain and the UK, respectively. In both cases, the contribution of demographic and socio-economic drivers, particularly age and gender, is broadly in line with the past evidence on cultural participation. For screen media consumption in terms of watching TV, playing console games and internet surfing, Escardíbul et al. (2013) investigate survey data of secondary school teenagers in Spain and further find that consumption is influenced by peer effects. The authors also identify statistically significant gender effects with the peer effects appearing to be more significant for boys. These differences might conceivably have been exacerbated after the pandemic shock as schools were closed and Students were home-schooled, so that physical classroom interactions were interrupted.

In a recent paper, Aguiar et al. (2021) look at the time spent in so-called leisure-luxuries, such as video gaming and other recreational computer activities, and the labour supply of young Americans. The authors find that innovation improvements in these leisure-luxuries account for the reduction in labour supply of young relative to old US males since the year 2004, a downward trend accelerated by the Great Recession and only partially inverted afterwards.

### The pandemic shock: income and time use in general economic studies

The pandemic has prompted several general economics studies on its impact on income and inequality and on time allocation based on the increase in working from home and the outcome of bargaining within the household over different resources and activities. We provide here a brief review of this literature with respect to those pandemic effects that may also influence cultural consumption and how this could vary across individuals based on their characteristics, the main focus of this paper.

For example, Crossley et al. (2021) provide evidence of the extent of labour market shocks in the UK following the pandemic, showing that income losses in April–May 2020 mainly followed from a decline in hours worked rather than employment losses. While government job retention schemes have mitigated the adverse effects on incomes, the decline in household earnings has unequally affected individuals, with those on lower incomes or in younger cohorts hit harder. While novel literature on the effects of the pandemic on cultural consumption is now beginning to emerge (see, Feder et al., 2021), to the best of our knowledge, our paper is the first to investigate, in a longitudinal setting, shifts in consumption in relation to a wide set of socio-economic and occupational characteristics.

Beyond the effect of income changes, our work also relates to the theory of time allocation between work and leisure first proposed in economics by Becker (1965) and Mincer (1962). Given that the majority of individuals we observe are either working (full-time, part-time, self-employed) or studying, the Becker time allocation model may help understand increases/decreases in the consumption of leisurely activities/house chores versus paid work and (given differences in consumption technology) the allocation of time across different types of content. For those who are on Furlough, Unemployed or Retired, the main issue to consider is more in terms of allocation of time between house chores and leisurely activities and across different forms of content consumption. Furthermore, the allocation may well vary across leisure activities—and heterogeneously across groups, as documented by Aguiar et al. (2021)

The shock to time availability, and the allocation between work and leisure/recreation, may have also been felt differently across individuals, as the same restrictions on social interactions, such as school, workplace, public gatherings and venue closures, may have had different impacts depending on personal life circumstances and household contingencies, e.g. living with others, child and elderly care, and gender. Also, while working from home saves time in terms of commuting, it also implies battling with house chores and, for those with children, dealing with home-schooling after school closures. For example, for the USA, Barrero et al. (2020) report survey evidence indicating that over 52% of the Employed worked from home during the pandemic compared with just above 5% before. This eventually translated into massive savings in commuting times and the authors show how the use of this additional time, especially across leisure and work, differs depending on whether childcare is needed. People also seem to report fewer hours worked but spread over a longer working day.

Similarly, the same shock may have impacted differently on different demographics. Using mobility data based on mobile phone geo-tracking for Italy, Portugal and Spain, Caselli et al. (2020) report more reduced mobility for women and the younger cohorts of the population. For the USA, Russell and Sun (2021) show that the pandemic may have had disproportionate negative effects on women, especially those with young children, in terms of labour market participation. For the UK, an ONS study addressed how people spent their time during the first lockdown between the 28th of March and the 26th of April.^18^ Income inequality seems to have been associated with specific working arrangements during the pandemic, with higher earners being more likely to work from home and lower earners more likely to work in the office. Lower incomes are also associated with less time for leisure. The ONS survey also reports a reduction in commuting time (again different across different income brackets). The gender gap in unpaid work, such as housework, childcare/adult care, however, seems to have reduced. Those with children spent more time doing childcare, albeit the older cohort (the grandparents?) spent less time in the same activity.

Finally, another interesting branch of economic theory related to this study is that on social interactions due to Becker (1974). In these “new home economics” models, the unit of reference is not the individual but the household, composed of more than one individual, bargaining to achieve an optimal allocation of work and leisure. In our case, this applies to the case of individuals living with others, such as a partner, children or housemates. In all these cases, individuals may have to bargain with the rest of the household in order to consume certain forms of cultural content, especially those that require a shared medium (TV set, PC, game console, broadband bandwidth, etc.), influencing the consumption decision of one specific content type over another.

With the above in mind, in this paper, we investigate the impact of the pandemic on cultural and creative consumption focusing on differences across socio-economic status, gender, age, employment status and living arrangements of individuals. Whenever possible, we try to compare and contrast our results with the relevant existing economic evidence.

## Data and definitions

### A national survey of creative and cultural consumption

We exploit a unique survey of cultural and creative consumption by UK individuals aged 16 years and older during the first national UK lockdown.^19^ The lockdown officially began on the 23rd of March 2020 and the survey was run over a fieldwork period consisting of six consecutive waves from April to May 2020 with the first wave running from the 9th of April to the 19th of April. In each wave individuals were asked about their consumption of CCCs over the past week, meaning that for the first wave answers refer to weeks starting on the 30th March and the 6th April.^20^

The survey asks questions about “remote” and online consumption with respect to several different forms of cultural and creative content. As already mentioned, we concentrate, in particular, on Music, Movies, TV, Games, Books, Magazines and Audiobooks. By remote consumption, we refer to that taking place through any of the following: downloading, online access (e.g. streaming) or online purchasing of physical media like books, CDs and DVDs.

Crucial to our purposes, in the first wave of fieldwork, individuals were also asked about their consumption of each of the above CCCs over the previous three months, the period from January to March 2020. This allows us to derive binary indicators denoting access to each of the seven types of content considered, for the period spanning January–March 2020 (the “Pre”) and the period corresponding to the fieldwork (the “Post”). Our empirical assessment on the changes in cultural consumption relies on differences taking place between these two periods due to the “exogenous” introduction of the lockdown. We are unfortunately unable to account for changes that may happened in the short period between the introduction of the national restrictions and the reporting period. However, in the (likely) case that changes occurred in this short unreported period go in the same direction of the changes in the reporting period, our estimated changes attributed to the lockdown would, in the worst-case scenario, be “conservative”.

### Measuring cultural consumption

We construct binary indicators of the reported consumption decisions with respect to each content *k* of the $K=7$ categories, irrespective of the mode of consumption (downloading, streaming, physical purchase). These indicators are defined for each category *k*, as:

1 $$\begin{aligned} \text {CCC consumption}_{ik}=d_{ik}= {\left\{ \begin{array}{ll} 1,&{} \text {if}\;\left( \sum _{s\in S_{k}}\hbox {d}_{isk}=1\right) \ge 1\\ 0, &{} \text {otherwise} \end{array}\right. } \end{aligned}$$

where $s \in S_{k}$ denotes any of the access modes related to content *k*. For example, in the case of Music, $d_{i,Music}=1$ for individuals reporting to have either downloaded or streamed Music, as well as purchased physical copies of CDs, and zero otherwise. In the same way, $d_{i,Books}=1$ for Books if individuals have read books either by purchasing a physical copy and/or by accessing an e-book version.

We also aggregate the content-level indicators defined above to derive an overall measure of consumption with CCCs, which we interpret as a simple measure of “variety” in consumption. Since variety is often associated with greater welfare and well-being, it is interesting to investigate whether consumers have responded to the pandemic by narrowing or diversifying their consumption choices. Variety is formally measured by the count of the different categories of content consumed by individuals in a specific period of time, again, irrespective of the mode of access:

2 $$\begin{aligned} \text {CCC consumption variety}_{i}=V_{i}=\sum _{k=1}^{7}(d_{ik}) \end{aligned}$$

**Fig. 1:**
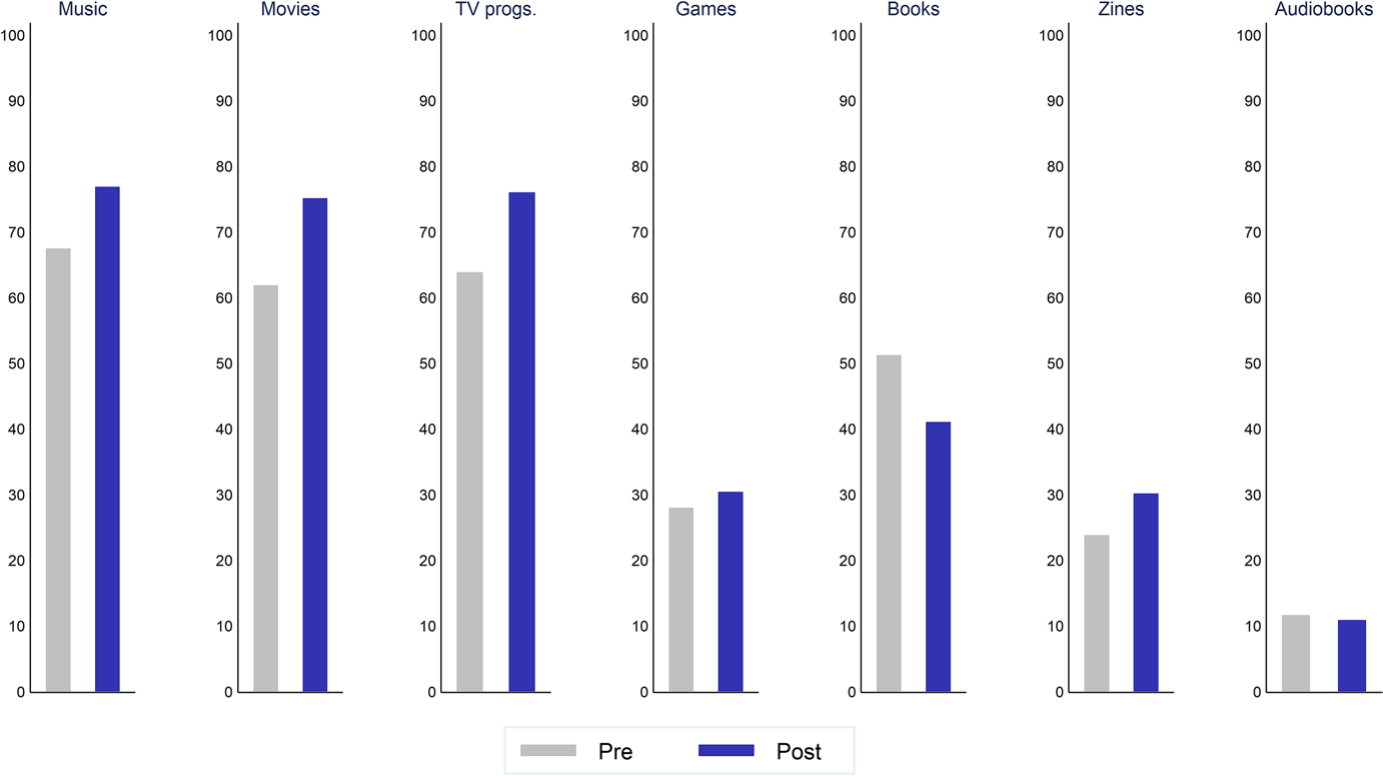
Consumption of different CCCs. The figure reports the percentage share of individuals consuming each content in the *Pre* and *Post* periods. All figures are weighted

Figure 1 contrasts the reported consumption during the fieldwork period (April–May 2020) with that during the previous three months (January–March 2020), reported in the first wave. Overall, the data show how Music, Movies and TV are the CCCs more largely consumed, and Books, Games, Magazines and Audiobooks are the ones with the lowest share of consumers.

**Fig. 2:**
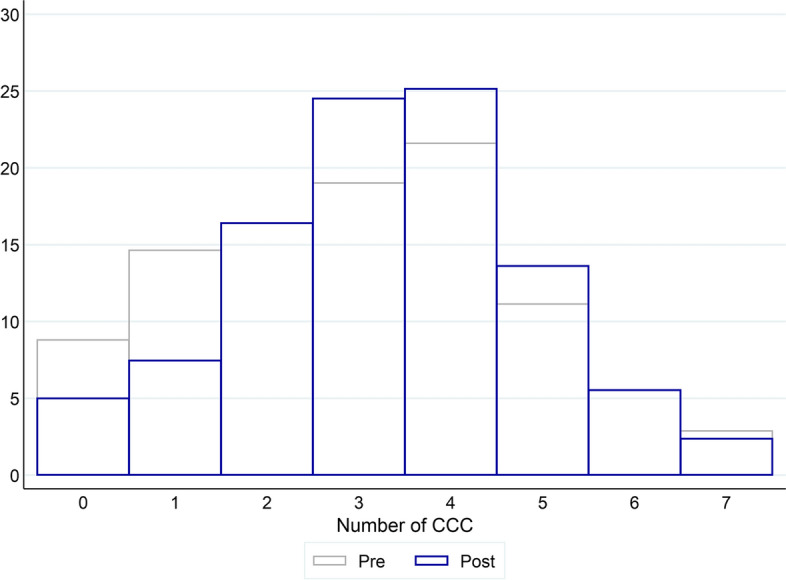
Overall consumption of CCCs. The figure contrasts the probability mass distribution for the variety of consumption measure (i.e. the count of content types individuals consume) in the *Pre* and *Post* periods. All figures are weighted

Comparison of the “Pre” and “Post” periods shows an overall increase for Music, Movies, TV programmes, Games and Magazines and a (small) reduction for Books and Audiobooks. We complement this picture by contrasting the “Pre” and “Post” variety in consumption with CCCs—as defined in Eq. (2). Figure 2 suggests an increase in variety, meaning an expansion in the set of CCCs respondents consumed. Such an expansion can be mainly attributed to a shift towards the central part of the distribution of consumption variety. At the same time, the share of highly consuming individuals, those consuming many types of content, stayed roughly constant across the two periods.

**Fig. 3:**
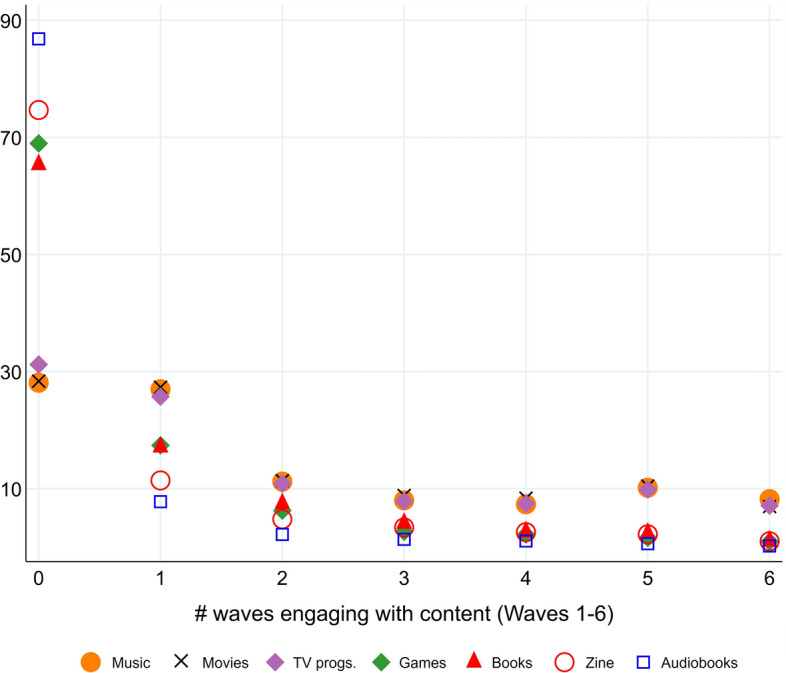
Frequency of consumption of CCCs. This picture reports individuals participation rates during the six waves of the fieldwork period (e.g. 87% of individuals never consumed audiobooks during the fieldwork period, etc.), separately for each content. All figures are weighted

Table 5 reports some summary statistics for each category of content, and the underlying modes of consumption, for the Pre and the Post periods, over the six waves of fieldwork (average and share of within-variance, that is, the fraction of overall variance accounted by changes reported by individuals over time—$\sigma ^{2}_{W}$—rather than between individuals—$\sigma ^{2}_{B}$). Beyond showing some differences in consumption across content categories (something we investigate in detail later), the table also provides additional insights. First, for each type of content, and associated access mode, the share of within-variance ranges from one third (Music) to a half (Books) of the overall variation over the fieldwork period, highlighting some sort of “switch” in consumption across individuals, a point which is confirmed in Fig. 3. Music, Movies and TV are the content categories exhibiting the highest share of individuals reporting to have consumed across all waves. Second, for all content types and modes of access, the individual participation rate, on average, between waves 1 and 6 is often lower—and in no case higher—than participation in the Pre period. This evidence suggests that it may be sufficient to contrast differences between the Pre and the entire *Post* period, which is what we will do later in the empirical analysis.

### Individual socio-demographic and economic characteristics

The survey data contain information regarding several socio-demographic and economic characteristics of the respondents. Here, we focus, in particular, on socio-economic status (SES), living arrangements, occupational status and demographics such as age and gender. The choice of these variables is broadly in line with what is considered in the literature reviewed earlier.

Household SES, in particular, is typically considered in the literature as a determinant of cultural consumption, especially in terms of the different consumption of highbrow and lowbrow activities. In relation to the effects of the pandemic, the aforementioned ONS reports indicate that the pandemic has hit harder households with lower SES. Hence, it is particularly interesting to look at how household SES is related to shifts during lockdown.

Since SES is not a uni-dimensional concept, in order to capture differences in SES across respondents, we construct a synthetic indicator via a Principal Components Analysis of the responses to two questions from the survey. The first replicates one of the questions in the National Readership Survey (NRS) on social grade, in particular the one related to the occupation of the main income earner in the household, and the second asks the income of the main household earner.^21^ We consider only the first principal component of the analysis (FPC, henceforth) since it accounts for almost 80% of the overall variation in the two variables (results are not reported for brevity but are available upon request). We then consider the quartile distribution of this indicator to capture group differences along the SES distribution.

Living arrangements capture whether respondents live alone or live with others. As mentioned earlier, since (Becker, 1974), the economics literature has already highlighted the role of bargaining within the household in the choice of time allocation. Some post-pandemic studies have discussed how living with others or caring for the elderly or children can affect time use (Russell & Sun, 2021). For example, watching TV and Movies can be a way of entertaining children stuck at home and spending family time together. However, it is also worth considering that some activities can trigger bargaining for the same medium, e.g. watching a specific TV programme or Movie or playing videogames if there is only one TV set or game console. There could also be some bargaining over the reading of Books or Magazines if these are shared, even though this should be less influential given that the cost of buying a second book/magazine is likely smaller than that of a new console or TV set. Also, while some media are compatible with doing other activities, e.g. listening to Music can be done while reading, working or doing house chores, other media should be more exclusive, e.g. watching TV should be less compatible in general with playing Games or reading Books or Magazines. To consider the effects of living arrangements, we include a set of dummies that distinguish between the following categories: living alone, living with children (w/children), living with a partner (w/partner), living with parents (w/parents), living with flatmates (w/mates).

Critical to understanding changes in cultural consumption is also the occupational status of respondents. For instance, individuals in employment face a leisure-work choice, unlike the Unemployed and the Retired (at least, with regard to formal occupations). In order to account for occupation-related differences, we consider binary indicators for the following categories: Employed, Unemployed, Retired and Students. However, while the data allow us to take into account of both individuals’ occupation and household SES, the survey does not ask individuals about their educational attainment. Finally, we consider age quartiles to capture different age cohorts and a gender dummy.^22^

Table 6 reports some key summary statistics for the SDEs considered. Respondents are almost equally split by gender and are, on average, fifty years old. The Employed account for 60% of the sample, with the Retired being the second largest category (24%). Nearly 20% of respondents live by themselves and 60% declare that they live with a partner. Furthermore, one-third of respondents live with children and 13% with parents or grandparents. Unsurprisingly, individuals in the latter group are younger (the median is about thirty-two years old) than the rest of the respondents. These statistics are broadly in line with what would be expected in a nationally representative survey and in line with those from the general population.^23^

Questions regarding the aforementioned characteristics are either asked in the first wave only or in all six waves. Table 6 shows that there is—as we would expect—a negligible variation of the SDE over the 6 waves. In the empirical analysis, then, we consider the self-reported SDEs from the first wave.^24^

## The Great Lockdown, cultural consumption and its micro-level characteristics

In this section, we turn our attention to investigating what is behind the evidence in Fig. 1. In particular, we test for the presence of statistically significant shifts in consumption of each of the different types of content considered and in the overall variety in consumption of CCCs. At the same time, we estimate the relationship between cultural and creative consumption and its micro-level determinants. We begin by looking at individuals’ decisions with respect to each of the seven types of CCCs using the following specification:

3 $$\begin{aligned} d_{ikt}=\mathbf {1}(\alpha _{0k}+\mathbf {\alpha _{1k}}\mathbf {x}_{i}+\theta _{k} {\textit{Post}}_{t}+\mathbf {h}_{ikt}+\varepsilon _{ikt}>0), \end{aligned}$$

where $d_{ikt}$ denotes indicator variables accounting for whether individuals consume content *k*, as defined in Eq. (1), **1**$(\cdot )$ denotes the indicator function and the coefficient $\theta _{k}$ captures the average change in the likelihood that respondents consume content *k* following the introduction of SiP measures (the “Post”); the parameter vector $\alpha _{1k}$ accounts for the differences in consumption with respect to the set of (observed) SDEs of interest, $\mathbf {x_{i}}$, and $\mathbf {h_{ikt}}$ additionally includes other observable confounding factors. These include (foreign) nationality, engagement in other online activities, frequency of internet use, region and region $\times$ Post fixed effects. The latter should capture baseline differences and post-lockdown differences across the twelve UK regions. Finally, $\varepsilon _{ikt}$ captures the unobserved characteristics and is distributed according to a logistic distribution. We cluster standard errors at the individual level, while all estimates are weighted by age gender region. As it stands, Eq. (3) aims to test the extent to which the introduction of SiP measures affected the likelihood of consuming each content *k*—after controlling for the described observable characteristics.

In the first seven columns of Table 1, we report the results from estimating Eq. (3) separately for each type of content. Reported estimates refer to the exponentiated-form coefficients (i.e. odds ratios), meaning that estimates tell us how changes in any of the explanatory variables impact the likelihood of consuming, i.e. $(d_{ikt}=1)$, relative to the alternative of non-consuming a specific type of content, i.e. $(d_{ikt}=0)$.^25^

Conditional on the observables, the results show a positive shift in consumption for Music and Games following the implementation of SiP measures. For both forms of content, the odds of consuming in the Post period were, on average, more than double those of the Pre period. In contrast, the corresponding estimated parameter for Books (which, to recall, includes both digital consumption and physical purchases) pointed to a decrease in the probability of consumption. The absence of statistically significant changes in consumption of Movies, TV programmes and other Publishing content (Magazines and Audiobooks) adds to the picture of content-specific changes. It also reflects the fact that, despite the shock, consumers seem to have maintained their consumption of creative and creative goods, which attests to their structural importance in the consumption basket.

Although the estimated $\theta$s in Eq. (3) can be thought of as reflecting changes in the share of individuals consuming CCCs, it is also worth considering these results in light of the existing content-level differences in terms of “popularity”, reflected in Fig. 1. The variations in consumption, instead, even within the narrowly defined time period under observation, can be observed also from Fig. 3.

**Table 1:** Determinants of cultural consumption: baseline estimates

	Dependent variable: $d_{ikt}$							Dependent variable: $V_{it}$
	Music	Movies	TV progs.	Games	Books	Magazines	Audiobooks	
Post	2.26**	1.44	1.21	2.32**	0.54**	1.35	1.03	1.15**
	(0.81)	(0.66)	(0.47)	(0.86)	(0.13)	(0.47)	(0.48)	(0.07)
*Occupational status* (Omitted cat: Retired)								
Employed	1.27	1.22	1.41	0.89	1.17	0.65*	2.23*	1.08
	(0.33)	(0.30)	(0.33)	(0.30)	(0.27)	(0.16)	(1.05)	(0.07)
Unemployed	0.79	1.07	1.07	1.75	1.02	0.68	1.02	1.04
	(0.26)	(0.32)	(0.31)	(0.67)	(0.29)	(0.21)	(0.62)	(0.08)
Student	1.00	2.16	3.74**	1.29	2.67**	0.50	2.51	1.23**
	(.)	(1.14)	(2.31)	(0.67)	(1.23)	(0.24)	(1.91)	(0.10)
*Living condition:* (Omitted cat: Living alone)								
w/children	0.91	1.89***	1.21	1.18	0.82	1.01	0.90	1.05
	(0.16)	(0.32)	(0.20)	(0.20)	(0.13)	(0.18)	(0.24)	(0.04)
w/partner	1.06	0.63**	0.79	0.95	1.23	0.82	1.13	0.97
	(0.21)	(0.12)	(0.14)	(0.18)	(0.21)	(0.17)	(0.32)	(0.04)
w/parents	0.94	0.81	1.28	0.92	0.95	1.72**	0.85	1.02
	(0.28)	(0.23)	(0.36)	(0.24)	(0.24)	(0.44)	(0.35)	(0.05)
w/mates	5.64**	1.52	1.99	0.69	1.76	0.73	2.17	1.14*
	(4.69)	(0.81)	(1.07)	(0.30)	(0.74)	(0.38)	(1.27)	(0.08)
*Age* (Omitted cat: Bottom quartile)								
2nd quartile	0.54**	0.52***	0.76	0.55***	1.15	1.98***	0.97	0.95
	(0.14)	(0.12)	(0.17)	(0.10)	(0.21)	(0.42)	(0.26)	(0.03)
3rd quartile	0.31***	0.37***	0.51***	0.31***	1.45*	2.37***	0.74	0.87***
	(0.08)	(0.09)	(0.12)	(0.07)	(0.28)	(0.54)	(0.24)	(0.04)
Top quartile	0.26***	0.32***	0.45***	0.26***	1.32	2.76***	1.15	0.85***
	(0.08)	(0.09)	(0.12)	(0.08)	(0.31)	(0.75)	(0.49)	(0.05)
*Household SES* (Omitted cat: Bottom quartile)								
2nd quartile	0.84	1.17	1.00	1.07	1.36	1.03	1.31	1.04
	(0.20)	(0.25)	(0.21)	(0.23)	(0.28)	(0.23)	(0.42)	(0.05)
3rd quartile	1.13	1.77**	1.45	0.65*	1.69**	0.96	0.86	1.08
	(0.32)	(0.44)	(0.34)	(0.16)	(0.39)	(0.24)	(0.32)	(0.05)
Top quartile	1.15	1.88***	1.60*	0.69	2.27***	1.04	1.33	1.12**
	(0.30)	(0.45)	(0.38)	(0.17)	(0.50)	(0.24)	(0.43)	(0.05)
Female	0.57***	0.83	0.84	0.42***	1.55***	0.92	0.75	0.93**
	(0.09)	(0.13)	(0.12)	(0.07)	(0.21)	(0.14)	(0.17)	(0.03)
Region f.e.	Yes	Yes	Yes	Yes	Yes	Yes	Yes	Yes
Region $\times$ Post f.e.	Yes	Yes	Yes	Yes	Yes	Yes	Yes	Yes
*N*	1756	1818	1818	1818	1818	1818	1818	1818
Log-L.	− 877.34	− 956.61	− 971.83	− 871.16	− 1155.01	− 1001.00	− 545.10	− 3297.54

Balanced sample. Weighted estimates. Standard errors clustered at individual level. Significance levels: *10%, **5%, ***1%. Results in all columns also control for frequency of access to internet, engagement with other online activities (e.g. through social media or for video meetings) and an indicator of foreign nationality. Household SES (and the associated quartiles) is defined based on the first principal component out of income and social grade for the main earner in the household. Higher values of the log-likelihood denote a better model fit

Turning our attention to the individual socio-demographic and economic characteristics of respondents, we can highlight, in particular, the statistically significant differences in consumption patterns related to age and gender. Respondents in the older cohorts in particular were less likely to consume Music, Movies, TV and Games than those in the younger cohort (the baseline category), and are more likely to read Books (albeit with only 10% statistical significance) and Magazines. No statistically significant age-related differences in consumption were found for Books or Audiobooks.

In terms of gender-related differences, female individuals were around 50% less likely to consume Music and Games and 50% more likely to consume Books. As discussed earlier, previous evidence mostly focuses on live attendance events and, as such, it may not be directly comparable with our results. However, the existing evidence for Music is mixed and also depends on the specific musical niche considered, as argued by Favaro and Frateschi (2007). Regarding Games, our results are consistent with the findings of Borowiecki and Prieto-Rodriguez (2015).^26^ We do not have directly comparable previous evidence for Books. However, to the extent that the consumption of Books falls within the category of highbrow activities, our evidence would be in line with studies documenting the prevalence of women in highbrow CCCs, like Mauri and Wolf (2020).

Turning our focus to the other SDEs, we find only small differences in consumption based on occupational status. Students were far more likely to consume Books and TV (between 2.7 and 3.7 times, respectively), compared with the baseline category of the Retired. The Unemployed were less likely to read Magazines and more likely to listen to Audiobooks (albeit with only 10% significance level).

By contrast, we can highlight some more substantive content-specific differences across household SES and living arrangements. In particular, the results are suggestive of a split in the likelihood of watching Movies and reading Books based on the household’s SES. Respondents who were part of a household in the third and top quartile of the distribution were between 1.7 and 2.3 times more likely to consume these forms of content than those in households at the other end of the distribution. Individuals from households in the top quartile of the SES distribution were also more likely to watch TV (albeit at the 10% level of statistical significance), and those in the 3rd quartile are less likely to play Games. Among the possible factors driving the results, access to capital goods may actually affect individuals’ consumption, hence making it easier—all else equal—for households in better economic circumstances.

Individuals with children were nearly twice as likely to watch Movies compared with those living alone, who are treated as the baseline category. Furthermore, this result is to be interpreted conditional on any living arrangement characterizing individuals with children (i.e. whether living with a partner, with their parents, etc.).^27^ This result is particularly interesting in light of the negative association, often discussed in the literature, between the presence of children in the household and cultural participation (see Ateca-Amestoy & Ugidos, 2021, Deaton et al., 1989, Muniz et al., 2014, among others). The “remote” nature of consumption can give a possible rationale for this result. However, this result also consists with the possibility that creative and cultural pursuits are the preferred way that parents entertain and spend time with their children. In contrast, living with a partner is associated with a lower likelihood of consuming Movies by about one-third. It may be important to read this result in relation to the fact that the baseline category refers to individuals living alone and that about half of the individuals living with a partner also have children in the household (hence, it should be considered jointly with the previously mentioned effect of children). Other differences concern the higher likelihood of consumption of some types of content, e.g. Music for those in shared living arrangements (w/mates) and Magazines for those living with their parents, and the lower likelihood of watching Movies for those living with a partner.

These results are robust to the inclusion of other factors that are typically investigated in the literature. For instance, unobserved consumption habits (whether framed in terms of addictive behaviour or learning by consumption) may affect the propensity to consume CCCs. We have tried to control for these effects by including the overall number of types of content consumed by individuals, as defined in Eq. (2). Also, gender-related differences may also be reflected in terms of bargaining within the household. Accordingly, we also account for this possibility by including an indicator of whether the main household earner is a woman. In both cases, the results are widely stable to these additional controls and, while not reported for brevity, they are available upon request.

The final column of Table 1 reports the results of a regression where variety, $V_{it}$, is considered as the left-hand variable. In this case, given that our measure of variety is a count variable, we use a Poisson estimator. Results show that consumers responded to the lockdown by increasing their variety of CCCs consumption in the Post period compared with the previous three months. To the extent that variety in consumption is also associated with well-being, this result may speak to the importance of culture and creativity in sustaining people during lockdown. As for the baseline association between variety and the SDE, we can see how several categories of consumers display a statistically higher association with a greater variety of CCCs’ consumption. Females, Students, those living with others (w/mates), those in the older cohorts of the population and those in the top quartiles of the SES distribution are all associated with a greater variety in consumption. The evidence on Females seems in line with previous literature on the gender difference in cultural consumption through more traditional cultural types of content. The result on SES could be in line with the evidence of inequality in the access to CCCs: individuals from households with higher status tend to consume a greater variety of content types.

It is not possible, however, from the above results, to gauge how shifts in consumption choices (and the overall variety) are associated with the list of SDE considered. We assess the latter in the next section.

## Shifts in consumption and SDEs

We now look for statistically significant shifts in consumption of each of the CCCs considered in relation to the set of SDEs of interest.

### Content-level consumption

We model shifts in consumption decisions by augmenting the specification in Eq. (3) with interactions of each SDE (labelled as **z** in what follows) with the Post indicator variable:

4 $$\begin{aligned} d_{ikt}=\mathbf {1}(\beta _{0k}+\beta _{1k}\mathbf {x}_{i}+\beta_{{\mathbf{2k}}}\mathbf {z}_{i}+\gamma _{k}\mathbf {z}_{i}\times {\textit{Post}}_{t}+\xi _{ikt}>0), \end{aligned}$$

The results from estimating Eq. (4) for each content *k* are reported in Table 2. Blocks from A to E present the interactions of each SDE with the Post indicator. In addition to the vector of characteristics already included in Table 1, whose baseline coefficients have now been omitted for brevity, each characteristic is in turn interacted with the ${\textit{Post}}$ indicator.^28^ Therefore, for each content type *k*, the estimated coefficient (vector) $\gamma _{k}$ in Eq. (4) tests for *Pre*–*Post* changes in the average likelihood of consumption that are specific to the SDE considered in turn, separately for each category each SDE consists of.

To gauge differences attributable to SiP measures, we test for statistically significant differences in the odds-ratio coefficients of Table 2. We report in Table 3 only the statistically significant differences in odds ratios. This reveals that the Retired were also more likely to play Games than the Employed. We do not find evidence of differences in consumption compared with the Unemployed. These differences inversely mirror within-lockdown consumption of Books for those Retired who, alongside the Employed, were less likely to access this type of content compared with Students and Unemployed.

We find moderately significant statistical evidence of changes associated with living arrangements. Namely, we can flag a smaller probability of consuming Books for those living alone and those living with a partner or their parents. Individuals living alone almost doubled their odds of consuming Games compared with the pre-lockdown period.

Interestingly, we do not uncover any significant differences in consumption for individuals living with children, either compared with the period preceding the introduction of SiP measures or in comparison with individuals in other living arrangements. However, this specific living arrangement could be compatible also with other living arrangements. Furthermore, it should be taken into consideration that in our sample the great majority of individuals living with children (about 80%) also live with a partner. We further investigate possible differences in consumption for these groups, restricting the analysis to the sub-sample of individuals living with a partner. Even in this case, we do not uncover any differences following the implementation of national restrictions.

**Table 2:** Determinants of cultural consumption: socio-economic channels

Dependent variable: $d_{ikt}$							
	Music	Movies	TV progs.	Games	Books	Magazines	Audiobooks
*Block A: Occupational status*							
Employed $\times$ Post	2.43**	1.41	1.19	1.99*	0.57**	1.49	0.95
	(0.88)	(0.68)	(0.47)	(0.76)	(0.15)	(0.57)	(0.44)
Unemployed $\times$ Post	1.79	1.28	0.95	2.47**	0.76	1.01	1.09
	(0.72)	(0.65)	(0.42)	(1.05)	(0.26)	(0.44)	(0.57)
Retired $\times$ Post	2.10*	1.51	1.30	3.60***	0.43***	1.28	0.91
	(0.85)	(0.70)	(0.57)	(1.65)	(0.12)	(0.46)	(0.56)
Student $\times$ Post	1.00	1.89	1.20	1.61	0.96	1.11	2.90
	(.)	(2.06)	(1.19)	(0.75)	(0.45)	(0.76)	(2.24)
*Block B: Living condition*							
Alone $\times$ Post	1.16	0.82	1.22	1.89*	0.58*	0.94	1.84
	(0.38)	(0.31)	(0.42)	(0.65)	(0.16)	(0.32)	(0.95)
w/children $\times$ Post	1.03	1.43	1.13	1.28	0.91	1.15	1.18
	(0.22)	(0.34)	(0.27)	(0.31)	(0.17)	(0.26)	(0.34)
w/partner $\times$ Post	1.27	1.24	1.35	1.43	0.44***	0.96	0.91
	(0.35)	(0.38)	(0.38)	(0.43)	(0.09)	(0.25)	(0.31)
w/parents $\times$ Post	1.13	0.85	0.52	1.22	0.56**	0.76	1.23
	(0.48)	(0.37)	(0.23)	(0.44)	(0.16)	(0.29)	(0.60)
w/mates $\times$ Post	0.77	1.95	2.12	2.10	0.71	1.38	1.86
	(0.76)	(1.46)	(1.70)	(1.04)	(0.28)	(1.02)	(0.99)
*Block C: Household SES*							
Bottom quartile $\times$ Post	2.29**	1.59	1.49	2.28**	0.75	1.32	1.29
	(0.90)	(0.78)	(0.66)	(0.92)	(0.21)	(0.48)	(0.68)
2nd quartile $\times$ Post	1.78	1.49	1.15	2.83***	0.53**	1.16	1.42
	(0.73)	(0.70)	(0.49)	(1.11)	(0.16)	(0.43)	(0.80)
3rd quartile $\times$ Post	2.34**	1.18	1.08	2.32*	0.53**	1.73	1.08
	(0.88)	(0.59)	(0.46)	(1.03)	(0.15)	(0.73)	(0.51)
Top quartile $\times$ Post	2.53**	1.66	1.19	1.80	0.44***	1.20	0.70
	(1.02)	(0.77)	(0.50)	(0.73)	(0.12)	(0.45)	(0.34)
*Block D: Gender*							
Male $\times$ Post	2.43**	1.49	1.12	2.19**	0.49***	1.25	0.84
	(0.91)	(0.70)	(0.46)	(0.82)	(0.13)	(0.47)	(0.42)
Female $\times$ Post	2.15**	1.42	1.28	2.53**	0.58**	1.44	1.33
	(0.80)	(0.66)	(0.50)	(1.00)	(0.15)	(0.50)	(0.62)
*Block E: Age*							
Bottom quartile $\times$ Post	4.35***	1.50	1.13	1.88	0.88	1.25	1.47
	(1.94)	(0.86)	(0.54)	(0.74)	(0.25)	(0.55)	(0.79)
2nd quartile $\times$ Post	2.13*	1.21	1.42	1.99*	0.64	1.14	0.80
	(0.83)	(0.59)	(0.63)	(0.79)	(0.18)	(0.47)	(0.42)
3rd quartile $\times$ Post	1.92*	1.25	1.00	2.21*	0.52**	1.08	1.15
	(0.70)	(0.55)	(0.39)	(0.92)	(0.14)	(0.39)	(0.58)
Top quartile $\times$ Post	2.33**	1.65	1.38	3.34***	0.46***	1.60	0.88
	(0.90)	(0.81)	(0.56)	(1.45)	(0.13)	(0.59)	(0.44)

Results from logistic regressions reporting exponentiated coefficients (i.e. odds ratios). Balanced sample. Weighted estimates. Standard errors clustered at individual level. Significance levels: *10%, **5%, ***1%. Results in all columns also account for baseline levels of SDEs as in Table 1

Worthy of mention are also the differences due to household socio-economic standing (block C), where we see some interesting results. For example, we observe a generalised increase in the likelihood of consuming Music over the SES distribution (with the exception of the second quartile). We find evidence of significantly increased consumption of Games across all household SES but the top quartile. The opposite occurs in terms of the observed slowdown in Books consumption, where individuals that are part of households in the bottom SES quartile are the only ones not recording a statistically significant reduction compared with the Pre-lockdown period. Books are in fact the only category where we can detect some asymmetry in consumption during lockdown compared with the Pre period: the estimated odds of consuming more during lockdown for individuals at the bottom of the SES distribution turn out to be statistically different from those at the top. Apart from this particular finding, the empirical evidence suggests, by and large, the absence of asymmetries in the likelihood of consuming CCCs during lockdown based on SES, i.e. pre-existing differences in consumption due to socio-economic inequality persisted also following the implementation of SiP orders.^29^

**Table 3:** Testing differences on “Post” coefficient

Content	SDE/category	$\chi ^2$(1)	*p*-value
*Occupational status*			
Games	Employed = Retired	3.07	0.0799
	Retired = Student	3.40	0.0652
Books	Unemployed = Retired	3.74	0.0532
	Retired = Student	3.20	0.0739
Audiobooks	Employed = Student	3.64	0.0565
*Living condition*			
Movies	Live alone = Live w/partner	2.73	0.0983
TV progs.	Live alone = Live w/parents	4.46	0.0347
	Live w/children = Live w/parents	3.08	0.0793
	Live w/partner = Live w/parents	6.65	0.0099
	Live w/parents = Live w/mates	2.76	0.0967
Books	Live w/children = Live w/partner	6.18	0.0129
*Household SES*			
Books	Bottom quartile = Top quartile	5.42	0.0200
Audiobooks	Bottom quartile = Top quartile	4.24	0.0394
	2nd quartile = Top quartile	3.15	0.0760
*Gender*			
Audiobooks	Female = Male	3.60	0.0579
*Age*			
Music	Bottom quartile = 2nd quartile	4.36	0.0369
	Bottom quartile = 3rd quartile	5.66	0.0173
	Bottom quartile = Top quartile	3.30	0.0691
Games	Bottom quartile = Top quartile	3.27	0.0704
4cmBooks	Bottom quartile = 3rd quartile	5.77	0.0163
	Bottom quartile = Top quartile	7.78	0.0053
Audiobooks	Bottom quartile = 2nd quartile	3.78	0.0518

This table reports statistically significant differences within SDEs coefficient estimates reported in Table 2

Turning our attention to gender differences, the observed average changes in consumption seen in the “Post” coefficients of Table 1 are essentially replicated when estimating separate coefficients for male and female respondents. For both genders, we find an increased (double) likelihood of consuming Music and Games but a decreased (half) likelihood of consuming Books. While our previous findings highlighted some gender differences in the overall consumption of CCCs, the shifts following the implementation of SiP measures are not statistically different between males and females.^30^

Finally, we can flag differences in the odds of consuming across age cohorts in relation to Music and Games (increase), as well as Books (decrease). While respondents across all age groups increased their odds of listening to Music compared with the preceding period, we find that the youngest were significantly more likely to consume this specific content than all other groups. Games consumption shows a somewhat opposite pattern, with higher odds of playing games for the older cohorts. This result is consistent with the earlier evidence on the increased average consumption of Games for the Retired. Also, testing for within-lockdown differences in consumption highlights asymmetries between groups at the extremes of the age distribution, a pattern which holds more strongly for Books, where we find that individuals at the high end of the age distribution are significantly less likely to consume compared with the pre-lockdown period and compared with the younger cohorts (during lockdown).

Overall, these results highlight a good deal of heterogeneity in the observed shifts in relation to specific SDEs and with respect to the observed changes in consumption of Music, Games and Books, except for differences in living arrangements. But even these effects are not even. When we investigate the extent of within-lockdown asymmetries in consumption across SDEs, we uncover that these exist primarily in relation to age, and secondarily in relation to the occupational category and living arrangements.

### Shifts in consumption variety

We have seen from Fig. 2 how during lockdown there was in general an increase in overall variety of CCCs consumed. After controlling for the SDE and the additional confounding factors, the last column of Table 1 confirms that consumption variety on average increased in the Post period. In this section, we aim to explore shifts in the variety of consumption of CCCs in relation to the characteristics of individuals. To this end, while we use a specification similar to that in Eq. (4), we need to account for the count nature of the left-hand side, our measure of consumption variety $V_{it}$ set in Eq. (2). Accordingly, we estimate a set of Poisson regressions. Results are presented in Table 4.

The first column reports, for comparison, the Post coefficient from the last column of Table 1. Interestingly, when we focus on Pre–Post changes, we see that the increase in consumption variety during lockdown is consistent across all SDEs, except for the category of living arrangements. Within the occupational status categories, Students are the only group not recording an increase in variety during lockdown compared with the Pre period, a result possibly reflecting an already diversified pre-existing consumption. However, when we test for differences in the coefficients reported in Table 4, no statistically significant differences emerge (again, results are not reported for brevity but are available upon request).

Focusing on within-lockdown changes, we also find that individuals living with their parents experienced a lower variety in consumption of CCCs compared with other living arrangement categories. In assessing this result, it is worth considering that this group of individuals mostly consists of those in the younger cohort.

It is also noteworthy that individuals from households at the top end of the SES distribution did not experience any increase in variety following the implementation of SiP measures. Furthermore, when testing for within-lockdown differences, we find statistically significant evidence of an increase in the variety of CCCs consumed for individuals in the bottom household SES quartile, as opposed to those at the other end of the distribution. Such evidence further contrasts with that suggesting that inequality of access to culture for individuals from poorer backgrounds became more pronounced in the pandemic. If anything, the evidence we gather—at least in the Great Lockdown—suggests the opposite.

**Table 4:** Overall consumption of cultural and creative content types

Dependent variable: $V_{i}$						
	(1)	(2)	(3)	(4)	(5)	(6)
Post	1.15**					
	(0.07)					
*Occupational status*						
Employed $\times$ Post		1.13**				
		(0.07)				
Unemployed $\times$ Post		1.15*				
		(0.10)				
Retired $\times$ Post		1.19**				
		(0.09)				
Student $\times$ Post		1.09				
		(0.09)				
*Living condition*						
Alone $\times$ Post			1.04			
			(0.06)			
w/children $\times$ Post			1.04			
			(0.05)			
w/partner $\times$ Post			1.03			
			(0.05)			
w/parents $\times$ Post			0.93			
			(0.06)			
w/mates $\times$ Post			1.06			
			(0.08)			
*Household SES*						
Bottom quartile $\times$ Post				1.23***		
				(0.09)		
2nd quartile $\times$ Post				1.15**		
				(0.08)		
3rd quartile $\times$ Post				1.14**		
				(0.07)		
Top quartile $\times$ Post				1.09		
				(0.07)		
*Gender*						
Male $\times$ Post					1.12*	
					(0.07)	
Female $\times$ Post					1.18***	
					(0.07)	
					(0.04)	
*Age*						
Bottom quartile $\times$ Post						1.12*
						(0.07)
2nd quartile $\times$ Post						1.10
						(0.07)
3rd quartile $\times$ Post						1.10
						(0.07)
Top quartile $\times$ Post						1.21***
						(0.08)
Region f.e.	Yes	Yes	Yes	Yes	Yes	Yes
Region $\times$ Post f.e.	Yes	Yes	Yes	Yes	Yes	Yes
*N*	1818	1818	1818	1818	1818	1818
Log-L.	− 3297.54	− 3297.27	− 3297.32	− 3296.42	− 3297.74	− 3296.51

Estimates from Poisson regression reporting exponentiated coefficients (i.e. incidence ratios). Balanced sample. Weighted estimates. Standard errors clustered at individual level. Significance levels: *10%, **5%, ***1%. Results in all columns also control for baseline levels of SDEs variables as in Table 1. Higher values of the log-likelihood denote a better model fit

## Summary and conclusions

In this paper, we use a unique longitudinal survey of UK adults designed to investigate consumption of cultural and creative forms of content during the implementation of national restrictions in Spring 2020, the “Great Lockdown”. We look, in particular, at remote or online consumption of cultural and creative types of content like Music, Movies, TV, Games, Books, Magazines and Audiobooks. Compared to past literature, then, we look at both a variety of content types that have not been previously considered and a different mode of consumption that traditional live-in-person.

We investigate the decisions of individuals to consume different types of content and also the overall variety of CCCs they consume, and test whether these changed in the Post SiP (during lockdown) compared with the Pre SiP period (before lockdown). Also, we look at the role of a number of socio-demographic and economic variables of interest, typically considered in the literature on cultural consumption, in determining cultural and creative consumption, irrespective of differences in this role before and after SiP measures. Then, we investigate the extent to which the observed shifts in remote and online cultural and creative consumption are specific to each of the socio-economic and demographic factors of interest.

First, our evidence points toward shifts in consumption decisions that are content-specific. In particular, within the set of CCCs we analyse, we find statistically significant evidence of an increase in the likelihood of consuming Music and Games and a reduction in the likelihood of consuming Books. We also find evidence of an increase in the variety of consumption.

In terms of the socio-demographic and economic characteristics of consumers, irrespective of difference between pre and post lockdown, we find that “popular” forms of content, like Games, Movies, Music, and TV were more common in the consumption baskets of the younger cohort of respondents and males. In contrast, content types related to Publishing were more likely to be associated with females (Books) and the older cohort of respondents (Magazines). We find little evidence of different living arrangements being associated with different CCCs consumption patterns in general, with the exceptions of those having children in the household being more likely and those living with a partner being less likely to consume Movies. Also, people in the higher quartile of the SES distribution are more likely to consume Movies and Books. While reading may arguably be viewed as a highbrow activity, watching movies may arguably be regarded along the same lines only for some types of film that might be considered highbrow. In comparison with the existing evidence on traditional access to culture, i.e. live attendances, it would be tempting to think of home “remote” consumption as more “horizontal” (what is sometimes referred to as a “democratising effect” of digitisation). It is also true, however, that access to Movies nowadays requires subscription services which can be expensive.

In order to assess the effects of the introduction of SiP measures in relation to the SDEs, we carry out a “Pre–Post” comparison exercise. We find some evidence of shifts in consumption of CCC for the majority of SDE groups, with little evidence for living arrangements, which perhaps suggests that the lockdown did not trigger changes in bargaining situation within the household. Possibly an exception is Books, where those living alone, those with a partner or parents displayed lower odds of consumption, which could suggest a shift away from less sociable activities in a period with forced social distancing.

We do not find evidence that enforced consumption from home increased pre-existing inequalities related to socio-economic status. The likelihood of consuming Music increased across individuals irrespective of their socio-economic standing, and there were no changes in the likelihood of consuming with other content types related to this characteristic, with the exception of Books and Games. With regard to the former, those at the top of the distribution were more likely to reduce the consumption of this type of content compared with those at the lower end of the distribution—a finding that is interesting if reading books is considered a highbrow activity. For the latter, a higher likelihood of consuming in the Post period is associated with lower socio-economic status.

We find more evidence of changes in consumption with specific types of content during SiP orders depending on the socio-demographic characteristics of the respondents. Other things equal, those in the older cohorts, and the Retired, were more likely to reduce Books consumption and to increase it with Games (compared with the younger cohorts).

When we consider shifts in the variety of consuming CCCs, we also find an increase in consumption in the Post period. This increase again varies with several socio-demographic and economic characteristics, with the notable—and surprising—exception of living arrangements. Among the other SDEs, the younger and the older seem to have increased the variety of their consumption, as well as females, those in higher SES status (with the exception of the highest group) and all occupational statuses with the exception of Students.

In assessing the scope of these results, it is important to bear in mind the limitations and drawbacks of the analysis. First, in estimating consumption decisions, we may be missing shifts in the intensity of consumption (e.g. the amount of time spent on CCCs). Second, obvious caveats apply to establishing causation from the estimated effects, as the objective to investigate the (time invariant) SDE drivers comes at the cost of overlooking individual unobserved heterogeneity, just to mention one obvious possible source of bias due to omitted factors.

In spite of these limitations, this work has the merit of contributing to the literature in a number of ways. It provides novel evidence on home and “remote” consumption of a wide range of cultural and creative types of content that has received less attention so far in the economics literature compared with live-in-person events. More importantly, since the implementation of shelter-in-place and social distancing due to the pandemic has forced individuals to spend more time at home and change their habits, this paper allows us to shed light on the shifts in behaviour during this period and how such shifts are related to personal characteristics. Our findings should be of interest to all readers concerned with understanding the effects of the Covid-19 pandemic and the future of cultural consumption in the new normal.

## Appendix

See Fig. 3 and Tables 5 and 6.

**Table 5:** Consumption of CCCs

	*Pre-*	*Post (Waves 1–6)*	
	Share	Share	$\sigma ^{2}_{W}$
*Music*	0.65	0.53	0.33
(Download)	0.37	0.20	0.41
(Online)	0.59	0.48	0.34
(Phys. Copies)	0.15	0.06	0.36
*Movies*	0.63	0.52	0.37
(Download)	0.31	0.19	0.45
(Online)	0.54	0.46	0.37
(Phys. Copies)	0.18	0.08	0.46
*TV progs.*	0.59	0.50	0.37
(Download)	0.26	0.16	0.46
(Online)	0.53	0.45	0.36
(Phys. Copies)	0.05	0.05	0.44
*Games*	0.32	0.16	0.37
(Download)	0.23	0.14	0.38
(Online)	0.16	.	.
(Phys. Copies)	0.13	0.05	0.38
*Books*	0.48	0.20	0.42
(Download)	0.26	0.15	0.37
(Online)	0.10	.	.
(Phys. Copies)	0.34	0.09	0.52
*Zine*	0.24	0.17	0.37
(Download)	0.08	0.07	0.40
(Online)	0.06	.	.
(Phys. Copies)	0.17	0.12	0.38
*Audiobooks*	0.14	0.07	0.39
(Download)	0.11	0.06	0.40
(Online)	0.07	.	.
(Phys. Copies)	0.03	0.03	0.43

All figures are weighted. Column 1 reports the share of individuals reporting to have consumed in the *Pre*, while column 2 reports the average (across waves) share of individuals consuming in the *Post*. Column 3 reports the average share of within-individual variance (within-variance) in consumption across waves 1 to 6, with $\sigma ^{2}_{W}$ denoting the share of within-individual variance, that is: $\sigma ^{2}_{W}=\frac{\sigma ^{2}_{W}}{\sigma ^{2}_{W}+\sigma ^{2}_{B}}$

**Table 6:** SDE descriptive statistics

	Mean	$\sigma ^{2}_{W}$
*Main household income*		
Bottom	0.40	.
Bottom-mid	0.20	.
Upper-mid	0.15	.
Upper	0.25	.
*NRS social grade*		
C2	0.17	.
C1	0.32	.
B	0.25	.
A	0.07	.
Other	0.19	.
Age	48.67	.
Other online activities	7.11	.
Female	0.51	.
Non-British ethnicity	0.13	.
Access internet > once a day	0.96	.
*Occupational status*		
Employed	0.60	0.02
Unemployed	0.12	0.04
Student	0.04	0.02
Retired	0.24	0.03
*Living condition*		
Alone	0.19	0.02
w/children	0.33	0.02
w/partner	0.59	0.02
w/parents	0.13	0.03
w/mates	0.03	0.04

All figures are weighted. $\sigma ^{2}_{W}$ denotes the average within-individual variance, with: $\sigma ^{2}_{W}=\frac{\sigma ^{2}_{W}}{\sigma ^{2}_{W}+\sigma ^{2}_{B}}$. “Main household income” (distribution quartiles reported) and “NRS social grade” refer to the main earner in the household, where “Other” groups together social grades “D—Working class”, “E—Non-working” (see footnote 21 for further details). “Other online activities” is defined as the count over a number of activities (e.g. social media activities, video meetings, etc.) survey participants are asked about in relation to their consumption t prior to Covid-19 pandemic outbreak. In relation to occupational status, “Employed” individuals account for full-time and part-time employees, self-employed, individuals already declaring to be on furlough (who can belong to any of the previous working categories)

## Footnotes

1 For detailed information on the stringency of government measures and measures to support the economy, please refer to https://www.bsg.ox.ac.uk/research/research-projects/oxford-covid-19-government-response-tracker and https://www.imf.org/en/Topics/imf-and-covid19/Policy-Responses-to-COVID-19.

2 For data before the pandemic, https://www.ons.gov.uk/peoplepopulationandcommunity/personalandhouseholdfinances/expenditure/bulletins/familyspendingintheuk/april2018tomarch2019 and, for a focus on Covid19, see https://www.ons.gov.uk/peoplepopulationandcommunity/personalandhouseholdfinances/expenditure/bulletins/familyspendingintheuk/latest.

3 Both for-profits and non-profits organizations relied on digital platforms as a source, respectively, to generate/increase revenues or to accomplish their “public good creation” goal. See, for instance, Towse and Handke (2013) or Falk and Sheppard (2006).

4 See, among others, https://www.bpi.co.uk/news-analysis/fans-turn-to-music-to-get-through-2020-as-a-new-wave-of-artists-fuels-streaming-growth/ on Music, https://www.pec.ac.uk/policy-briefings/on-demand-culture-how-the-lockdown-is-changing-games-and-streaming-services on digital gaming and broadcasting platforms, and also https://www.culturehive.co.uk/wp-content/uploads/2021/09/COVID-19-and-the-Global-Cultural-and-Creative-Sector-Anthony-Sargent.pdf and https://pro.europeana.eu/post/the-digital-transformation-agenda-and-glams-culture24-findings-and-outcomes.

5 See, for instance, recent survey evidence from the USA: https://www2.deloitte.com/content/dam/insights/us/articles/6456_digital-media-trends-covid/DI_Digital-media-trends-14th-edition.pdf.

6 On this point, previous literature has shown that cultural consumption affects mental health outcomes (Crociata et al., 2014) and post-pandemic studies (see Tubadji, 2021) have found that those with greater cultural exposure pre-pandemic exhibited higher levels of well-being during the pandemic, making the case for the importance of sustaining the cultural industries also with public funding.

7 See, for example, Jonhson (2020) on the relationship between arts and culture in the “new normal”. Not specifically on cultural consumption, but more generally on consumption habits, Sheth (2020) discusses how some of the habits arising in response to the pandemic may persist afterward, in particular in reference to the use of digital.

8 https://pec.ac.uk/news/how-can-the-creative-industries-come-together-to-share-how-covid-19-is-impacting-the-sector.

9 https://pec.ac.uk/blog/cultural-consumption.

10 https://www.theaudienceagency.org/evidence/covid-19-cultural-participation-monitor. See also Johannes et al. (2022).

11 For a more general comparative analysis of time use during lockdown in the UK, see Roberts (2020).

12 For a timeline of the UK restrictions see https://www.instituteforgovernment.org.uk/sites/default/files/timeline-lockdown-web.pdf.

13 https://www.gov.uk/government/publications/full-guidance-on-staying-at-home-and-away-from-others.

14 While variety in cultural consumption can also be associated with cultural omnivorousness, the latter often refers to the consumption of both highbrow and lowbrow activities by higher class individuals (see literature the review section). In our case, our measure of variety does not make this distinction, so one has to be careful in associating increases in variety with increases in omnivorous behaviour.

15 For reviews, see Seaman (2005) and McKenzie and Shin (2020).

16 Again, the interested reader can refer to the reviews in these papers.

17 Within the set of “traditional” cultural consumption, exceptions to this mainly pertain to recorded music consumption, as in the already mentioned studies of Favaro and Frateschi (2007) and Prieto-Rodriguez and Fernandez-Blanco (2000).

18 For greater detail, see https://www.ons.gov.uk/economy/nationalaccounts/satelliteaccounts/bulletins/coronavirusandhowpeoplespenttheirtimeunderrestrictions/28marchto26april2020.

19 The survey instrument over the six weeks of the first lockdown builds on the pre-existing Online Copyright Infringement survey carried out by the UK Intellectual Property Office (IPO), and it is the result of a collaboration between the UK Creative Industries Policy Evidence Centre (https://pec.ac.uk), the IPO and AudienceNet (https://www.audiencenet.com). An extension of the survey was performed later, thanks to funding by UKRI over three more waves after the relaxation of the national lockdown and during a period of local lockdown measures. In this paper, we concentrate on the first national lockdown, using the first six waves of the survey.

20 Fieldwork dates concerning the following waves (2nd to 6th) are, respectively: 20th–26th April, 27th April–3rd May, 4th–10th May, 11th–15th May, 16th–20th May.

21 In particular, NRS social grade is established by asking about (main household earners) individuals’ occupation, as follows: “A—Upper middle class”, “B—Middle middle class”, “C1—Lower middle class”, “C2—Skilled working class”, “D—Working class”, “E—Non-working”. Please see https://www.nrs.co.uk/nrs-print/lifestyle-and-classification-data/social-grade/ for further details.

22 The results do not change if for age, as an alternative to the quartile distribution, we consider a binary-median-based indicators or the continuous variable.

23 For greater detail, please refer to the census: https://www.ons.gov.uk/peoplepopulationandcommunity/populationandmigration/populationestimates.

24 Unfortunately, we are unable to account for those re-allocations that may have taken place in the short period between the beginning of lockdown and the reporting period of the first wave. This does not change the nature of our hypotheses with respect to the relationship between consumption choices and SDE during lockdown.

25 Therefore, a coefficient greater (smaller) than one would reflect an increase (decrease) in the likelihood of consumption relative to the alternative of non-consuming.

26 Borowiecki and Bakhshi (2018) also show that females are less likely to play games, although, conditional on playing, they seem to be doing it more frequently than males.

27 For the sake of clarity, all living arrangement categories but those accounting for the presence of children are strictly mutually exclusive. At the same time, while individuals living, for instance, with their partner may also have children in the household, the presence of children in the household is nonetheless mutually exclusive with respect to the baseline category of those living by themselves.

28 The results are generally stable to estimating Eq. (4) after including the full set of SDEs interactions with the *Post* indicator.

29 We have also investigated differences in terms of the two variables underlying the indicator of household SES taken one at the time, i.e. household income and NRS social grade of the main earner in the household, with no substantial differences in results. Again, these results are not reported but are available upon request.

30 The same holds when investigating differences in terms of the main household earner being female (results not reported but available upon request).
